# Catal-GPT: AI-driven directed efficient design framework for catalysts

**DOI:** 10.1093/nsr/nwaf299

**Published:** 2025-07-25

**Authors:** Peng Zheng, Zhennan Han, Bao-Lian Su, Guangwen Xu

**Affiliations:** Key Laboratory on Resources Chemicals and Materials of Ministry of Education, Shenyang University of Chemical Technology, China; Key Laboratory on Resources Chemicals and Materials of Ministry of Education, Shenyang University of Chemical Technology, China; State Key Laboratory of Advanced Technology for Materials Synthesis and Processing, Wuhan University of Technology, China; Laboratory of Inorganic Materials Chemistry (CMI), University of Namur, Belgium; Key Laboratory on Resources Chemicals and Materials of Ministry of Education, Shenyang University of Chemical Technology, China

## Abstract

This perspective article proposes and further preliminarily verifies an artificial intelligence assistant (Catal-GPT) that interacts with researchers to optimize catalyst formulations, thereby improving the efficiency of catalyst design.

The rapid development of Generative Pre-training Transformer (GPT) has found widespread applications across multiple research domains, including its implementation in catalysis research. With the improvement of data quality and model generalization ability, GPT-driven approaches are enabling more efficient and accurate catalyst development processes. This perspective paper explores and discusses the application potential of the qwen2:7b large language model (LLM) in catalyst design, which is used to extract the knowledge and generate the formulation suggestions for the oxidative coupling of methane (OCM) reaction [1], showcasing a transformative approach for accelerating catalyst development.

Nowadays, efficient catalysts can extensively reduce energy consumption and production costs while minimizing the generation of by-products and residues. Traditional catalyst design often relies on a trial-and-error approach and high-throughput experimentation. The trial-and-error approach is a common empirical exploration process that involves synthesizing and testing various materials to find candidates with the desired catalytic properties. This time-, resource- and labor-consuming method unfortunately lacks clear guidance, making the research and development process both costly and inefficient. Although trial-and-error methods can sometimes promote important discoveries, they struggle to systematically optimize catalyst performance or predict their behavior.

The involvement of GPT technology is bringing new opportunities for catalyst design, new protein [2]/drug structure [3] and material discovery [4–6]. This artificial intelligence (AI)-based technology utilizes deep learning models to understand and generate natural language text. In the field of catalyst development, GPT technology can be used to extract knowledge from vast amounts of scientific literature, experimental data and reaction mechanisms. Through training, the GPT model can comprehend complex chemical concepts and the implicit relationships within language, thereby predicting new catalyst combinations and design strategies. It can be seen that recent advances in LLMs have boosted the transformative progress in materials science, particularly in information extraction, decision-making and human–AI interactions. A growing body of literature demonstrates how LLMs are redefining traditional research rules by integrating multimodal data, enhancing autonomous reasoning and collaborating with human experts.

In the field of biomedical nanoscience, the development of the NanoSafari tool has represented a significant advancement in automated knowledge extraction [7]. This tool employed an innovative information extraction method based on Grouped Iterative Validation, which systematically extracted contextual information on nanoparticle characteristics from over 20 000 published articles. LLMs have also demonstrated strong capabilities in driving decision-making within complex material discovery processes. CrystaLLM was a pioneering example, utilizing an autoregressive LLM based on the Crystallographic Information File format to showcase the effectiveness of LLMs in generating credible crystal structures [5]. More recently, LLMs were being positioned as the central ‘dynamic coordinators’ within the framework of General Material Intelligence reported by Yuan *et al.*, where the core lay in their interaction and collaboration with human researchers [8].

The design principle of the Catal-GPT tool is based on a highly specialized system architecture, aimed at optimizing the design and screening process of industrial catalysts (Fig. 1). The system is constructed in a modular way, comprising data storage, foundation model, agent and feedback learning modules. The workflow initially involves collecting extensive datasets concerning catalyst synthesis, characterization and application, followed by cleaning and encoding these data to fit the input format of the model. Subsequently, a customized GPT model is trained to generate new catalyst structures, predict their performance, and suggest improvements based on specific catalyst design issues. In each iteration, the system learns from experimental results or industrial application feedback, continuously optimizing its recommendation strategy. This dynamic workflow allows researchers to explore the vast chemical space more efficiently, taking into account cost, efficacy and environmental impact, thereby driving catalyst development towards more efficient directions: customized industrial catalyst development and catalytic performance prediction etc.

**Figure 1.**
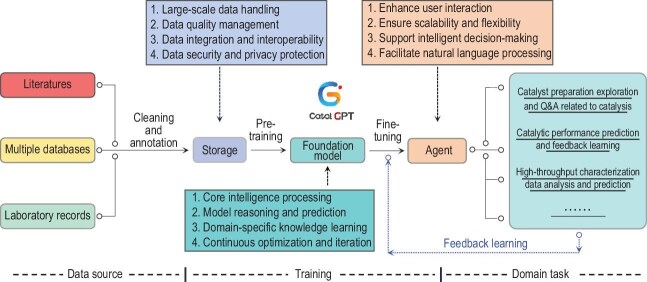
Schematic of the Catal-GPT workflow.

The existing specialized LLMs in catalysis and chemistry, including ChemCrow (specializing in organic synthesis, drug discovery and materials design), ChemLLM (focused on molecular nomenclature conversion, property prediction and reaction outcome forecasting) and ChemReasoner (integrating quantum chemistry for catalyst discovery) primarily address analytical or predictive tasks. In contrast, Catal-GPT establishes a possible paradigm as a natural language processing (NLP)-driven system dedicated to generating executable catalyst preparation methods, offering a unique pathway for catalyst development not covered by the existing benchmarks.

In this perspective article, the open-source qwen2:7b LLM with a database (doi numbers are provided in Table S1) on the oxidative coupling of methane reaction is employed for local deployment to verify the possibility of developing the professional GPT tools (Catal-GPT). The information annotation and dataset splitting methods can be found in the Supplementary data (Table S2). In the data preprocessing step, when conflicting parameters are detected for the same catalytic system, priority would be given to the preparation parameters that appeared in the authoritative publications with the highest reported frequency.

For the researchers, being able to directly ‘converse’ with the literature references to acquire needed knowledge, or to retrieve and analyze relevant data can significantly improve work efficiency. Therefore, the potential application of the qwen2:7b LLM in Catal-GPT development was analyzed through task evaluations, such as knowledge extraction and research assistance. It should be noted that the collected datasets related to catalyst synthesis, characterization and application underwent data cleaning to adapt to the model's input format before being submitted to the model. This step ensured the cleanliness and consistency of the input data, thereby improving the performance and predictive accuracy of the model. Figure 2a provides examples of knowledge extraction using qwen2:7b LLM. The accuracy of knowledge extraction rate was calculated to be 92% (Fig. 2b). The top-k recall analysis (Fig. 2c) revealed a significant divergence by question type. In Fig. 2c, k defines the tolerance of evaluation, which allowed the model to include the correct answer within its top-k most probable answers. It could be seen that the information extraction about the theoretical calculation methods could achieve 80% recall (top-1) and 100% recall (top-7), while the information about the catalyst preparation details exhibited relatively lower recall (plateauing at 80% by top-7). Additionally, research assistance tasks were also carried out on the preparation of OCM catalysts, and the results showed that the GPT model could propose a complete catalyst preparation process and can further refine the details of catalyst preparation process in subsequent questions (Fig. S1). It was evident that the qwen2:7b LLM can adapt well to knowledge extraction and research assistance tasks. Based on the results, it is considered that fine-tuning of the models is necessary in improving the reliability of producing desired outputs and correcting failures to follow the complex prompts. In the future, we aim to facilitate transferability to other catalytic systems through decoupling the generic domain knowledge from specific reaction parameters. Several challenges still need to be addressed before the migration process. For example, there is a need for real-time recognition of reaction types to adjust knowledge weighting, due to the differences in core parameters among the various catalytic systems. In addition, a term disambiguation library is also needed due to the input data formats varying significantly between the different catalytic systems. To facilitate broader validation efforts, we have made the source code (can be found in ‘source code.py’ in the Supplementary data) publicly available. This will enable researchers who possess high-quality data on the other catalytic systems to perform the relevant tests in the future.

**Figure 2.**
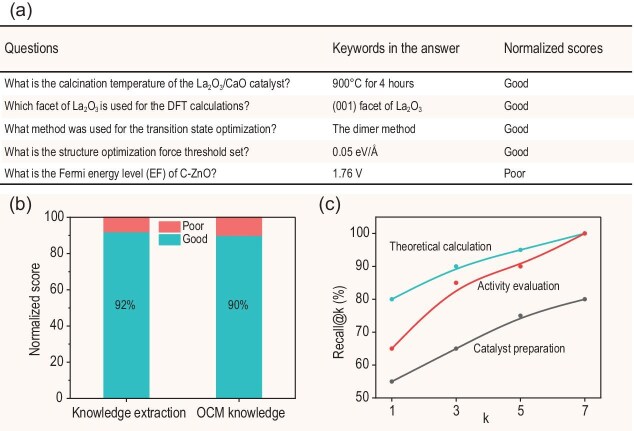
(a) Examples of knowledge extraction task by qwen2:7b. (b) Results of the responses of qwen2:7b to different tasks. (c) Top-k recall for the OCM catalyst preparation process knowledge extraction.

Therefore, it could be seen that by training a customized LLM, the system could extract knowledge from a vast amount of scientific literature and experimental data, and propose the design strategies of the catalyst. For example, in the Q&A section, the model was able to accurately answer the professional questions about the calcination temperature of the La_2_O_3_/CaO catalyst, the La_2_O_3_ crystal face used for the DFT calculations, and the structural optimization force threshold etc., showing its deep understanding and application ability of the data. The model not only proposed a complete preparation process for the OCM catalyst but also listed the required chemical reagents and their applications in detail, proving its practicality in catalyst design and research assistance. Furthermore, the model could predict new catalyst preparation processes based on literature references and refine the preparation details in the subsequent questions. The application prospects of Catal-GPT in the field of catalyst research are broad, and it is expected to promote technological innovation and development in this area.

Despite offering powerful data analysis and predictive capabilities in catalyst research and development, Catal-GPT still faces a range of challenges and limitations. One significant challenge is the quality and availability of data. The efficient operation of Catal-GPT relies on a vast amount of accurate experimental data, which often needs to be obtained through precise experiments. Therefore, ensuring data quality and proper data preprocessing is crucial. Another limitation is the model's generalization capability. Although Catal-GPT can discover complex relationships between data through machine learning algorithms, these models may struggle to make accurate predictions when faced with significantly different chemical systems. Moreover, there is a potential issue of overfitting, where the model adapts too much to the training data and loses its predictive power for new data.

To relieve the issues of insufficient data quality, noise interference and limited data availability, we expect to solve these issues through combining automated verification with data-sharing mechanisms, wherein the latter could expand the foundation of high-quality data. In detail, the standardized interface should be created; meanwhile, a data exchange standard for the catalytic field should be defined, thereby lowering the barriers to data-sharing. For the generalization, and overfitting, the use of more diverse catalytic datasets for pre-training will be explored to further enhance the universality of the foundational model. Moreover, more advanced regularization schemes will be systematically evaluated and compared. It is believed that these concrete future strategies provide a clear roadmap for addressing the current limitations of the current research.

In the future, several directions for Catal-GPT will be worth exploring in depth: a hybrid modeling framework based on dynamic prompt engineering can be developed, deeply coupling the semantic parsing capabilities of LLMs with physical models such as density functional theory calculations and molecular dynamic simulations, to construct enhanced intelligent agents with cross-scale reasoning abilities. In addition, the establishment of standardized knowledge graph construction protocols are considered to be necessary, enhancing the accuracy of unstructured experimental data (such as the reaction conditions) extraction by introducing self-supervised contrastive learning mechanisms. The technological breakthroughs in Catal-GPT will shorten the development cycle of catalysts and provide theoretical support for building the intelligent catalyst development platform.

## Supplementary Material

nwaf299_Supplemental_Files
