# Supplementary material for: Catal-GPT: AI-driven directed efficient design framework for catalysts
Source: Natl Sci Rev. 2025 Jul 25;12(9):nwaf299. doi: 10.1093/nsr/nwaf299 (PMC12421571; doi:10.1093/nsr/nwaf299)
Supplement: nwaf299_Supplemental_Files [file nwaf299_supplemental_files.zip › Supplementary data.pdf]

Electronic Supplementary Information

Catal-GPT: AI-Driven Directed Efficient Design Framework for Catalysts

Peng Zheng [a], Zhennan Han [a], Bao-Lian Su [b, c *], Guangwen Xu [a, *]

[a] Key Laboratory on Resources Chemicals and Materials of Ministry of Education, Shenyang University of Chemical Technology, Shenyang 110142, China

[b] State Key Laboratory of Advanced Technology for Materials Synthesis and Processing, Wuhan University of Technology, Wuhan 430074, China

[c] Laboratory of Inorganic Materials Chemistry (CMI), University of Namur, 61 rue de Bruxelles, Namur 5000, Belgium

Corresponding Author: *E-mail: baoliansu@whut.edu.cn (B. Su); bao-lian.su@unamur.be (B. Su); gwxu@ipe.ac.cn (G. Xu)

Contents

Contents 2

S1. DOI numbers of the references used in the evaluation of qwen2:7b 3

S2. Information annotation and dataset splitting 4

S3. Example of designing an OCM catalyst by qwen2:7b 6

S4. Configuration file (config.py) 7

S5. Source code (source code.py) 8

S1. DOI numbers of the references used in the evaluation of qwen2:7b

Entry	DOI	Entry	DOI
1	10.1016/j.jechem.2022.05.013	26	10.1021/acscatal.2c04637
2	10.1016/j.jechem.2021.01.008	27	10.1016/j.cej.2022.141057
3	10.1016/j.jechem.2023.12.025	28	10.1002/anie.202108201
4	10.1021/acscatal.3c06301	29	10.1021/acscatal.9b01585
5	10.1021/acscatal.0c04104	30	10.1002/anie.202117201
6	10.1021/acscatal.2c03747	31	10.1021/acscatal.9b01585
7	10.1021/acscatal.1c03496	32	10.1021/acscatal.1c05177
8	10.1021/acscatal.9b00022	33	10.1002/anie.202310525
9	10.1021/jacs.3c10069	34	10.1007/s11426-023-1766-8
10	10.1021/acscatal.4c02103	35	10.1021/acs.est.3c01941
11	10.1021/acscatal.2c03725	36	10.1002/anie.202007557
12	10.1021/acs.chemrev.6b00715	37	10.1021/acscatal.1c02315
13	10.1002/smll.202310857	38	10.1021/acscatal.0c01493
14	10.1016/j.apcatb.2021.120161	39	10.1039/d4ta02049a
15	10.1021/acscatal.1c04915	40	10.1002/anie.202304301
16	10.1021/acscatal.1c01392	41	10.1021/acscatal.0c03896
17	10.1021/acscatal.3c04714	42	10.1021/acscatal.9b03066
18	10.1016/j.cej.2023.141379	43	10.1021/cs501733r
19	10.1021/acscatal.9b05591	44	10.1016/S1872-2067(20)63756-1
20	10.1002/anie.202004778	45	10.1021/acscatal.3c05094
21	10.1002/anie.202319192	46	10.1021/acscatal.4c03001
22	10.1021/acscatal.8b04728	47	10.1016/j.jechem.2021.10.018
23	10.1021/acscatal.0c04888	48	10.1021/jacs.2c06143
24	10.1021/acscatal.3c05534	49	10.1021/acscatal.2c04916
25	10.1016/j.apcatb.2021.120553	50	10.1021/acscatal.1c04206

S2. Information annotation and dataset splitting

This research centered the evaluation criteria on a relatively streamlined measure of accuracy, which was defined as the proportion of correctly extracted knowledge units, such as the catalyst components or the reaction conditions etc., from the documents in the test set.

The information annotation was performed through the following steps.

Firstly, two researchers highlighted key paragraphs in the literature and performed initial extraction. In this step, the initial structured annotation of the information would be extracted. The example of the extraction of key information categories could be found in **Table S2**.

Table S2. The categories of key information.

Categories	Annotation contents	Examples
Catalyst supports	Support name (including composite support)	CaO etc.
Active components	Active metals/oxides loaded on supports	La_2O_3 etc.
Theoretical calculations	Methods, Software, and key conclusions	VASP etc.
Characterizations	Methods, Parameters, and key conclusions	XRD etc.
Catalytic activities	C_2 yield, selectivity, and CH_4 conversion	C_2 yield

Note: The information extracted from the same PDF document along with the DOI numbers will be placed in a single txt file.

Subsequently, the annotation consistency was ensured through cross-checking, where the conflict cases were verified through a review of the full-text context, such as the

symbol-type conflicts or numerical-type conflicts, etc.

The dataset splitting was carried out through the following steps.

Firstly, the collected information on the OCM catalysis was divided into three groups, which were rotated to serve as the test set. It was necessary to clarify that the similar catalytic systems (at least ensuring that the active components were similar) were evenly distributed into the different groups. Additionally, the year of literature publication was also taken into account within each group. This approach could avoid the problem of the model being only good at extracting outdated methods due to data timeliness deviations, but unable to capture the latest developments in the field (such as the application research of single-atom catalysts).

S3. Example of designing an OCM catalyst by qwen2:7b

QUESTION:

Based on the references, predict a new oxidative coupling of methane catalyst preparation process. Please list the required experimental chemicals in a table (Reagents and Applications). Additionally, provide a detailed preparation method.

ANSWER:

(1) Required Experimental Chemicals

The preparation of a novel catalyst for the oxidative coupling of methane involves several chemical reagents with specific functions:

REAGENT NAME	APPLICATION IN SYNTHESIS
$La(NO_3)_3$ $6H_2O$	Catalyst precursor
$CaCO_3$	Supports
$BaCl_2$	Provides an alternative surface for doping
NH_4OH	Used for ammonia vapor generation in N-doping

(2) Detailed Preparation Method

Step 1:

Dissolve $La(NO_3)_3$ $6H_2O$ in a sufficient volume of water to prepare a solution with the desired concentration. Add $CaCO_3$ slowly under stirring while ensuring the pH is maintained around 8 for successful precipitation of La_2O_3/CaO.

Step 2:

After the slurry has settled or been mixed sufficiently, spread it on a ceramic plate. Dry at around 100 °C to remove water and then calcinate under controlled conditions in an air atmosphere up to 800 °C for several hours to convert $CaCO_3$ into CaO.

Step 3:

If doping with BaX (where X = F or Cl) is planned as suggested in the context, mix La_2O_3/CaO and $BaCl_2$ powders under a mild grinding process. Calcinate this mixed material at high temperature to form new phases.

Step 4:

Generate ammonia (NH_3) by reacting NH_4OH with steam in the presence of O_2 gas for a short time, ensuring sufficient exposure to La_2O_3/CaO catalyst. The NH_3 molecules will diffuse into the catalyst matrix during calcination at high temperature, leading to N-doping.

Step 5:

Before use in the methane oxidative coupling process, activate the catalyst with hydrogen gas or oxygen gas under controlled conditions. This step ensures that surface active sites are prepared for effective reaction.

Figure S1. Example of designing an OCM catalyst by qwen2:7b.

S4. Configuration file (config.py)

Before running **source code.py** file, the settings could be configured according to the config.py file. And the **config.py** file could be found in the attachments. The detail is listed as follows:

```python
import os
from dotenv import load_dotenv

# Load environment variables
load_dotenv()

# API Configuration
# To get your API key:
# 1. Visit https://dashscope.aliyun.com/
# 2. Sign up/Login to your account
# 3. Go to "API Keys" section
# 4. Create a new API key or copy existing one
# 5. Replace the value below with your actual API key
DASHSCOPE_API_KEY = "your-api-key-here"
API_ENDPOINT = "https://dashscope.aliyuncs.com/api/v1/services/aigc/text-generation/generation"
# Model Configuration
MODEL_PATH = "sentence-transformers/paraphrase-multilingual-mpnet-base-v2"
# Qwen Model Configuration
QWEN_MODEL = "qwen2-7b-instruct"
QWEN_TEMPERATURE = 0.7
QWEN_TOP_P = 0.8
QWEN_SYSTEM_PROMPT = "You are a professional chemistry assistant."
# File Path Configuration
BASE_DIR = os.path.dirname(os.path.abspath(__file__))
DOCUMENTS_DIR = os.path.join(BASE_DIR, "txt")
KNOWLEDGE_INDEX_PATH = os.path.join(BASE_DIR, "knowledge_index")
# Text Splitting Configuration
CHUNK_SIZE = 500
CHUNK_OVERLAP = 50
# Retrieval Configuration
TOP_K_RESULTS = 3
# Display Configuration
DISPLAY_LENGTH = 80
# Offline Mode Configuration
OFFLINE_MODE = True
```

S5. Source code (source code.py)

The source code could be found in the source code.py file from the attachments.

The detail is listed as follows:

```python
# Install dependencies: pip install requests python-dotenv langchain sentence-transformers faiss-cpu

import os
import requests
import numpy as np
import faiss
import logging
from langchain_community.document_loaders import DirectoryLoader
from langchain.text_splitter import RecursiveCharacterTextSplitter
from sentence_transformers import SentenceTransformer
from config import *

print("Enabling detailed logging to monitor download progress")
logging.basicConfig(level=logging.INFO)
print("Loading configuration")

if OFFLINE_MODE:
    os.environ['TRANSFORMERS_OFFLINE'] = '1'
    os.environ['HF_DATASETS_OFFLINE'] = '1'

def load_documents(folder_path):
    loader = DirectoryLoader(folder_path, glob="**/*.txt")
    docs = loader.load()
    splitter = RecursiveCharacterTextSplitter(
        chunk_size=CHUNK_SIZE,
        chunk_overlap=CHUNK_OVERLAP
```

```python
    )
    chunks = splitter.split_documents(docs)
    return [chunk.page_content for chunk in chunks]

class KnowledgeBase:
    def __init__(self):
        print("Initializing KnowledgeBase")
        self.encoder = SentenceTransformer(MODEL_PATH)
        print("Initializing faiss")
        self.index = faiss.IndexFlatL2(768)
        print("Initialized faiss")
        self.documents = []

    def build(self, folder_path):
        texts = load_documents(folder_path)
        print("Texts: {}".format(texts))
        embeddings = self.encoder.encode(texts, show_progress_bar=True)
        self.index.add(np.array(embeddings))
        print("Indexed: {}".format(self.index))
        self.documents = texts

    def retrieve(self, query, top_k):
        query_embed = self.encoder.encode([query])
        distances, indices = self.index.search(query_embed, top_k)
        print("\nRetrieval Results Details:")
        print("-" * 50)
```

```python
        for i, (distance, idx) in enumerate(zip(distances[0], indices[0])):
            print(f"Similarity Score: {1/(1+distance):.4f}")
        return [self.documents[i] for i in indices[0]]

def qwen_chat(context, question):
    headers = {
        "Authorization": f"Bearer {DASHSCOPE_API_KEY}",
        "Content-Type": "application/json"
    }

    prompt = f"""Based on the following knowledge:
            {context}

        Please answer this question in English: {question}"""

    data = {
        "model": QWEN_MODEL,
        "input": {
            "messages": [
                {"role": "system", "content": QWEN_SYSTEM_PROMPT},
                {"role": "user", "content": prompt}
            ]
        },
        "parameters": {
            "temperature": QWEN_TEMPERATURE,
            "top_p": QWEN_TOP_P,
            "result_format": "message"
        }
    }

    try:
        response = requests.post(API_ENDPOINT, json=data, headers=headers)
        response.raise_for_status()
        return response.json()["output"]["choices"][0]["message"]["content"]
    except Exception as e:
        return f"API call failed: {str(e)}"

def chat_loop():
    print("Knowledge Q&A system started (type 'exit' to quit)")
    while True:
        query = input("\nPlease enter your prompt: ")
        if query.lower() in ["exit", "quit"]:
            print("System: Goodbye!")
            break
        contexts = kb.retrieve(query, TOP_K_RESULTS)
        print(f"\nNumber of retrieved contexts: {len(contexts)}")
        print("\nExtracted contexts:")
        for i, context in enumerate(contexts, 1):
            print(f"\nContext {i}:")
            print(f"{context[:DISPLAY_LENGTH]}...")
            response = qwen_chat(context, query)
            print(f"\nAnswer based on context {i}:")
            print(f"{response}")
            print("-" * 50)

if __name__ == "__main__":
    kb = KnowledgeBase()
    if not os.path.exists(KNOWLEDGE_INDEX_PATH):
        kb.build(DOCUMENTS_DIR)
        faiss.write_index(kb.index, KNOWLEDGE_INDEX_PATH)
    else:
        kb.index = faiss.read_index(KNOWLEDGE_INDEX_PATH)
        kb.documents = load_documents(DOCUMENTS_DIR)

    chat_loop()
```
